# IL-6/gp130 signaling: a key unlocking regeneration

**DOI:** 10.1186/s13619-023-00160-z

**Published:** 2023-03-23

**Authors:** Ruopu Li, Deqiang Li, Yu Nie

**Affiliations:** 1 State Key Laboratory of Cardiovascular Disease, Fuwai Hospital & Cardiovascular Institute, National Center for Cardiovascular Disease, Chinese Academy of Medical Sciences and Peking Union Medical College, 167 Street, Beilishi Road, Xicheng District, 100037 Beijing, People’s Republic of China; 2 The Center for Cardiovascular Research, the Research Institute at Nationwide Children’s Hospital, 575 Children’s Drive, Columbus, OH 43215 USA; 3 The Department of Pediatrics, College of Medicine, Ohio State University, Columbus, OH 43215 USA; 4 National Health Commission Key Laboratory of Cardiovascular Regenerative Medicine, Fuwai Central-China Hospital, Central China Branch of National Center for Cardiovascular Diseases, 450046 Zhengzhou, China; 5 Shenzhen Key Laboratory of Cardiovascular Disease, Fuwai Hospital Chinese Academy of Medical Sciences, 518057 Shenzhen, China

## Abstract

Liver is an organ with notable capacity of regeneration. Reprogramming of hepatocytes towards an immature state is one of the important mechanisms for hepatocyte replenishment. Inflammatory response mediated by IL-6 and its family cytokines has been widely reported closely related with tissue regeneration in myriads of organs. Recently Hui and colleagues reported that the dedifferentiation of hepatocytes depends upon IL-6 signaling from Kupffer cells and the reprogramming of gene expression under the inflammatory condition is different from the regulation of gene expression during embryo hepatocyte specification, highlighting a tight linkage between extracellular microenvironment and parenchymal cell plasticity during tissue regenerative repair.

## Main text

Following periportal injury, mature hepatocytes can undergo reprogramming and dedifferentiate into liver progenitor-like cells (LPLCs), which gain proliferative potential and repopulate the injured site. However, it has been an outstanding question how LPLCs are generated and what signaling cues instruct this process. A recent study from Hui and his colleagues uncovered surprising findings that Kupffer-cell derived Interleukin-6 (IL-6) is repurposed for LPLC derivation (Li et al. 2023a).

IL-6 family cytokines consist of IL-6, IL-11, IL-27, IL-31, oncostatin M (OSM), leukemia inhibitory factor (LIF), ciliary neurotrophic factor (CNTF), cardiotrophin-1 (CT-1) and cardiotrophin-like cytokine factor 1 (CLCF1) (Li et al. 2023b). The cytokines are grouped as one family since they share a common co-receptor gp130, which mediates trans membranous signaling transduction during acute tissue inflammatory response (Li et al. 2023b). The signaling pathways downstream of gp130 including JAK-STAT3, Src-YAP, and Notch signaling etc., all of which have been revealed closely related with tissue repair and regeneration.

Earlier, Hui and his colleagues unveiled that reprogramming of the hepatocytes to express progenitor-state genes was controlled by a permissive chromatin state established by Arid1a (Li et al. 2019). The permissive state of the chromatin can be considered as a preparation state of the cell to start up regenerative response under certain conditions, where genes associated with liver progenitor cells are continuously accessible but are not expressed under steady states and are expressed to endow the cell with immature profiles under certain stimulation.

Recently, Hui’s group showed that the anatomical distribution of reprogrammed LPLCs after liver injury was closely associated with the distribution of activated Kupffer cells neighboring portal veins and IL-6/STAT3 pathway bridging the Kupffer cell activation and LPLC formation and clarified the role of inflammation in bridging tissue damage and regeneration (Li et al. 2023a).

In adult homeostatic tissues, the population of cells is set to be at a certain level to control the organ size. Howerver, when tissue injury occurs, cell loss should be timely sensed to repopulate the cells at injured site through proliferation and differentiation. Impaired cells and cell contents released from the damaged cells are indicators of tissue damage and cell loss. Inflammatory cells, especially tissue resident macrophages sense the signal of cell loss and release inflammatory mediators, the subsequent signal, to instruct the parenchymal cells to switch on their regenerative response (Li et al. 2023b; Li et al. 2020a, 2020b).

In this model, loss of hepatocytes in tissue damage is sensed by and activate Kupffer cells surrounding portal veins. Activated Kupffer cells release IL-6 and trigger the STAT3 pathway in their neighboring hepatocytes. Here, IL-6 provokes the dedifferentiation of hepatocytes rather than its known role as inflammatory signal. Although the pro-regenerative role was repeatedly confirmed in the regeneration of other organs, elucidating the sentinel role of tissue resident macrophages in mediating tissue regeneration may allow us to better understand the mechanisms of how immune microenvironment affects organ homeostasis.

**Fig. 1:**
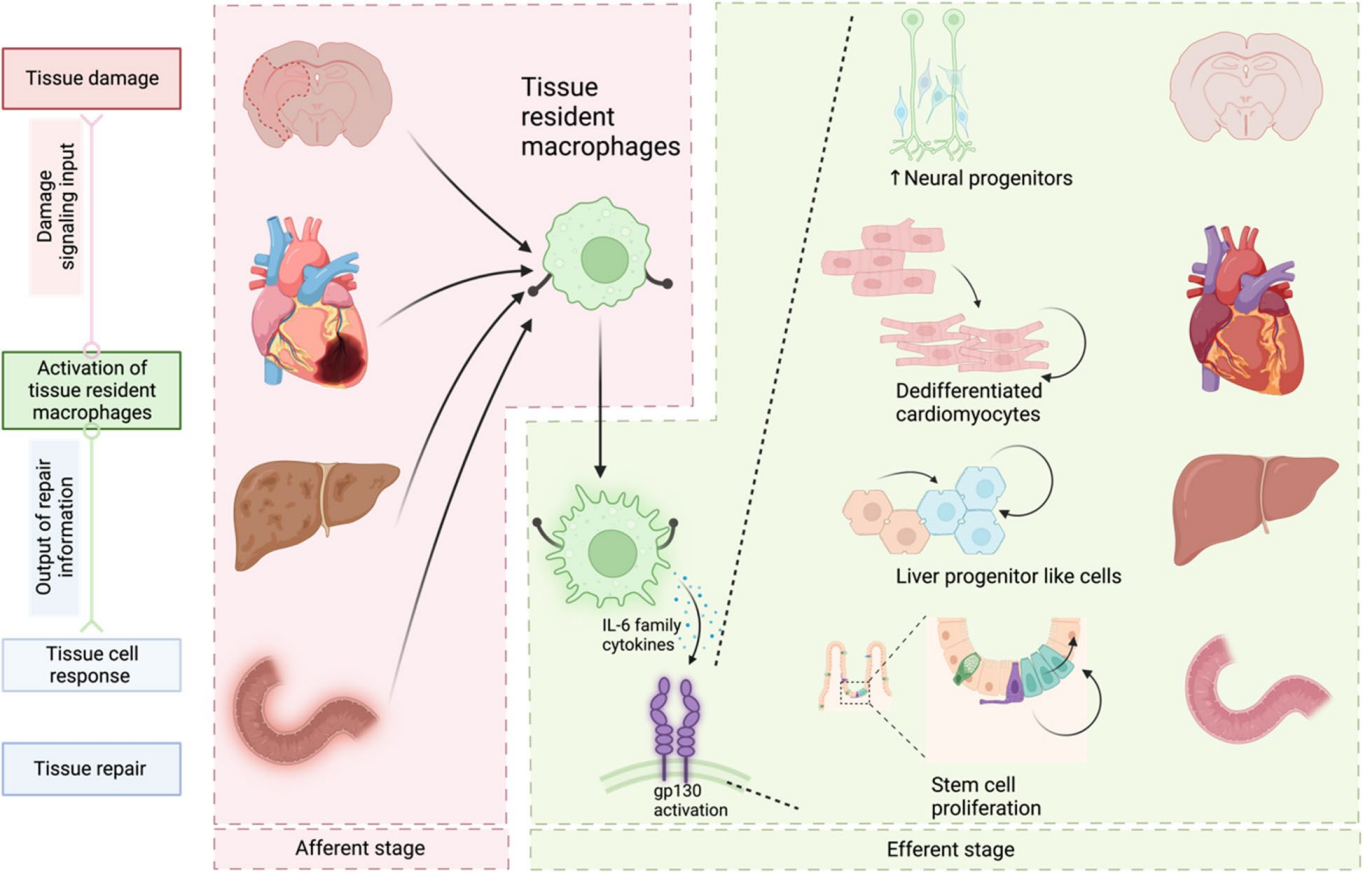
Tissue resident macrophages (TRMs) act as sensors of tissue injury. Damage signaling is sensed by and activates TRMs. Activated TRMs crosstalk with tissue cells by releasing IL-6 and its family cytokines to promote parenchymal reprogramming and provoke the proliferation capacity of the cells, leading to regenerative repair through the supplement of lost cells in tissue damage (Taniguchi et al. 2015; Li et al. 2020b; Willis et al.^ 2020^; Li et al. 2023a)

Series of studies revealed the role of IL-6 family cytokines and the co-receptor of all these cytokines gp130, in regeneration. Neonatal hearts temporarily hold the capacity to regenerate after cardiac injury. Han et al. reveals that acute inflammation is mandatory for the initiation of such repair mode and loss of IL-6/STAT3 signaling blocks heart regeneration (Han et al. 2015; Wang et al. 2019). IL-11 mediates the reprogramming of fibroblasts, endothelial cells, and parenchymal cells in the regeneration of zebra fish heart, fin and scale (Allanki et al. 2021). OSM triggers the dedifferentiation of cardiomyocytes, which is prerequisite for cardiomyocytes to re-enter cell cycle of proliferation (Li et al. 2020b). Olson and colleagues conducted single cell transcriptome RNA sequencing (scRNAseq) in non-myocytes in regenerating hearts and identified CLCF-1 as a strong stimulus to increase the proliferation of cardiomyocytes (Wang et al. 2020). In inflammatory hepatic tumor, several mutations of gp130 have been discovered and the mutation showed auto activation without ligands (Rebouissou et al. 2009). The activation of gp130 and its downstream signaling Src-YAP is activated after mucosal erosion of mouse small intestine and persistent activation of the pathway boost epithelial proliferation (Taniguchi et al. 2015). Also, terminally differentiated cardiomyocytes scarcely proliferate but their proliferation capacity was rekindled when the activated mutant of gp130 was expressed in cardiomyocytes (Li et al. 2020b). Gp130 activation in the central nervous system also promoted neuronal survival and neuroprotection via IL-6 tran-signaling mechanism, where gp130 was dimerized and activated through IL6 binding to its soluble IL-6 receptor (Willis et al. 2020). These observations suggest that gp130 signaling activated by IL-6 and its family cytokines may probably act as a key and shared pro-proliferation pathway mediated by post injury inflammatory response in multiple tissues and organs across the whole body (Fig. 1).

Reprogramming of hepatocytes induced by IL-6/STAT3 pathway was absent in hepatocyte during normal development. This emphasizes the difference between tissue regeneration and development and leads us to reconsider that the process of reprogramming the somatic cell to re-express immature genes such as reprogramming/progenitor-related genes (RRGs). Reprogramming can be achieved not only by simply replicating the gene expression mechanisms in early development but also by inducing inflammatory-mediated transcriptomic regulation. Hepatocytes reserved the permissive chromatin state allowing the inflammatory IL-6 signaling to provoke and reprogram in mature hepatocytes, demonstrating an induction role of cell microenvironment in regulating the stemness of parenchymal cells. By determining whether such chromatin permissive state of progenitor-like genes may be a promising way for us to explain the difference in reprogrammability of different lineages.

## Conclusions

In all, immune microenvironment interacts actively with tissue cells after injury and the interaction together present as the tissue response to injury. Inflammation senses the injury and output signaling in form of cytokines and chemokines. These factors subsequently regulate secondary reactions of other cells against injury. Different inflammatory paradigm represents different interaction among inflammatory cells, stromal cells and parenchymal cells and thus result in distinct outcomes of wound repair. IL-6 mediated inflammation has been discussed for years concerning its role in tissue repair. And whether the reported mechanism by Hui and colleagues is involved in the repair of other tissues remains further elucidation and more work from different fields are required to draw a comprehensive picture of IL-6/gp130 mediated tissue regeneration.

## Data Availability

Not applicable.
